# Discovering the genetic modules controlling root nodule symbiosis under abiotic stresses: salinity as a case study

**DOI:** 10.1111/nph.18627

**Published:** 2022-12-04

**Authors:** Jawahar Singh, Oswaldo Valdés‐López

**Affiliations:** ^1^ Facultad de Estudios Superiores Iztacala, Laboratorio de Genómica Funcional de Leguminosas Universidad Nacional Autónoma de México Tlalnepantla Estado de México 54090 Mexico

**Keywords:** GmNAC181, GmNSP1a, legume nodule symbiosis, legumes, salt stress, soybean, transcription factors

## Abstract

Legumes form a symbiotic association with rhizobia and fix atmospheric nitrogen in specialized root organs known as nodules. It is well known that salt stress inhibits root nodule symbiosis by decreasing rhizobial growth, rhizobial infection, nodule number, and nitrogenase activity in diverse legumes. Despite this knowledge, the genetic and molecular mechanisms governing salt stress's inhibition of nodulation and nitrogen fixation are still elusive. In this Viewpoint, we summarize the most recent knowledge of the genetic mechanisms that shape this symbiosis according to the salt levels in the soil. We emphasize the relevance of modulating the activity of the transcription factor Nodule Inception to properly shape the symbiosis with rhizobia accordingly. We also highlight the knowledge gaps that are critical for gaining a deeper understanding of the molecular mechanisms underlying the adaptation of the root nodule symbiosis to salt‐stress conditions. We consider that filling these gaps can help to improve legume nodulation and harness its ecological benefits even under salt‐stress conditions.

## Introduction

Members of the legume family (Fabaceae) engage in endosymbiosis with nitrogen‐fixing soil bacteria collectively known as rhizobia. This symbiosis leads to the formation of specialized root lateral organs called nodules. Inside these organs, rhizobia, in exchange for plant‐derived carbon‐rich organic compounds, convert atmospheric nitrogen into forms that the plant host can assimilate (i.e. ammonium) (Roy *et al*., 2020). Through root nodule symbiosis, legumes not only fulfill their nitrogen needs but also replenish the soil with fixed nitrogen. Indeed, legumes fix 60 million metric tons of nitrogen world‐wide every year (Smil, 1999), and this makes the root nodule symbiosis a climate‐smart alternative to reduce our agricultural dependency on synthetic fertilizers.

The model legumes *Medicago truncatula* and *Lotus japonicus* have been used to identify several genes involved in rhizobial infection and nodule formation, including a variety of transcription factors (TFs) (Chakraborty *et al*., 2022). Among them, CYCLOPS, through the action of DELLA proteins, forms a large complex with the two GRAS domain TFs Nodulation Signaling Pathway 1 (NSP1) and NSP2 to activate the expression of the *Nodule Inception* (*NIN*) TF gene (Oldroyd & Long, 2003; Hirsch *et al*., 2009; Jin *et al*., 2016). In turn, NIN fine‐tunes the expression of genes participating in rhizobial infection, nodule development, regulation of nodule number per root, symbiosome development, and the onset of nitrogen fixation (Hirsch *et al*., 2009; Cerri *et al*., 2012; Soyano *et al*., 2013; Liu *et al*., 2019, 2021). Because of these roles, NIN is considered a central regulator of root nodule symbiosis.

## NSP1: the first genetic component affected by salt stress

Root nodule symbiosis is negatively affected by different abiotic factors, such as soil salinity and drought. Salt stress inhibits root nodule symbiosis in diverse legumes by reducing rhizobial infection, the number of nodules, nodule weight, and nitrogenase activity, which all directly reduce the nitrogen fixation process (Singleton & Ben, 1984; Chakraborty *et al*., 2021). Despite this knowledge, we are still far from understanding the complete genetic mechanisms that modulate root nodule symbiosis under salinity conditions. However, recent research on soybean (*Glycine max*) has begun to fill this gap. For instance, it has been demonstrated that glycogen synthase kinase 3 (GSK3)‐like kinase (GmSK2‐8) inhibits rhizobial infection and nodule formation in soybean under salt‐stress conditions (He *et al*., 2021; Singh & Verma, 2021). Further experimentation demonstrated that GmSK2‐8 phosphorylates the phosphosites S212 and T214 located in the LHR1 domain of GmNSP1a. This phosphorylation inhibits GmNSP1a capability to bind to the promoter region of the symbiotic gene *GmERN1a*. Additionally, the overexpression of GmSK2‐8 significantly reduces the expression of *GmNINb* (an ortholog of *MtNIN*) and *GmENOD40‐1*. To this date, we can speculate that the reduction in the rhizobial infection process and nodule development under salt‐stress conditions can be due to: (1) a disruption in the formation of the CYCLOPS‐DELLA‐NSP2/1 complex inhibiting the transcriptional activation of *NIN*; or (2) the inability of the phosphorylated version of GmNSP1a in activating the expression of *GmERN1a* that could directly or indirectly inhibit the expression of *NIN*. In any of these hypotheses, the modulation of the spatiotemporal *NIN* expression and NIN activity seems to be crucial to shaping root nodule symbiosis according to salt levels in the soil.

## The interplay of NAC and NIN for root nodule symbiosis in soybean

**Fig. 1:**
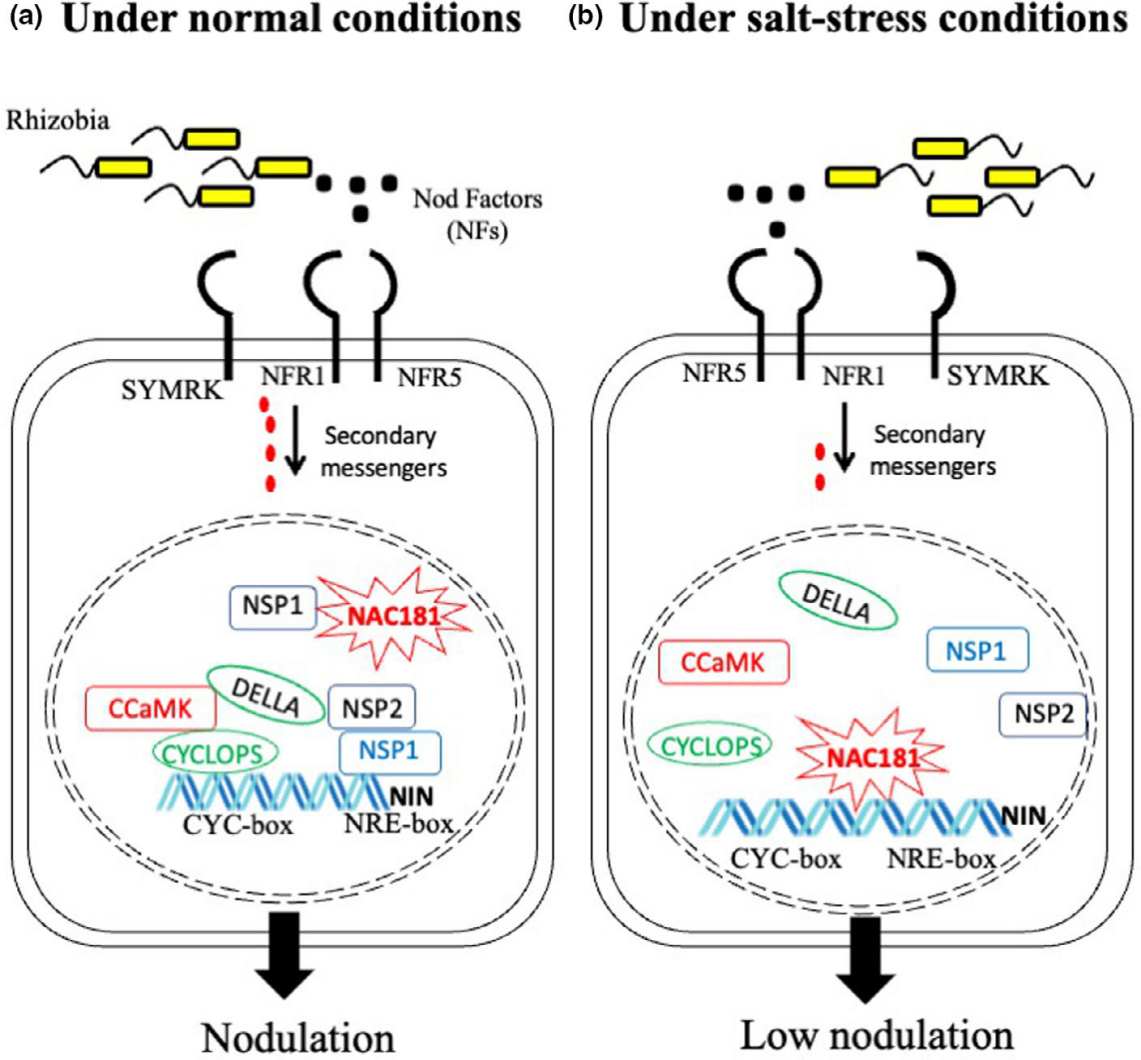
Conceptual model of GmNAC181 controlling salt stress‐induced *GmNINa* transcription for soybean nodulation. (a) Under normal conditions, a set of plasma membrane‐located receptor‐like kinases (i.e. NFR1, NFR5, and SYMRK) first recognizes Nod factors (NFs) from rhizobia to initiate the signaling cascade. Receptor complex at plasma membrane transduced signal from it to the nucleus by secondary messengers (i.e. mevalonate and calcium), activating CCaMK. Under normal circumstances, activated CCaMK phosphorylates CYCLOPS, which results in the formation of a bigger complex that includes DELLA, NSP1, NSP2, and likely GmNAC181. DELLA proteins serve as a link between NSP2–NSP1 and CCaMK‐CYCLOPS. The activation of this large transcription factor complex ensures the transcriptional activation of *NIN*. NIN, in turn, activates the expression of genes involved in each stage of the root nodule symbiosis. (b) Under salt stress conditions, the formation of the CYCLOPS‐DELLA‐NSP2/1‐NAC181 is disrupted, and very likely NAC181 could independently act to partially activate NIN. The partial activation of NIN could allow the legume host to form fewer nodules. NIN, Nodule Inception; NSP1, Nodulation Signaling Pathway 1; NSP2, Nodulation Signaling Pathway 2; GmNAC181, *Glycine max* N‐terminal DNA‐binding domain 181; NFR1, Nod factor receptor 1; NFR5, Nod factor receptor 5; SYMRK, Symbiosis receptor‐like kinase; CCaMK, calcium/calmodulin‐dependent protein kinase; CYC‐box, *cis‐*regulatory element recognized by the transcription factor CYCLOPS; NRE‐box, *cis‐*regulatory element recognized by the transcription factor NSP1. Differences in the thickness of black arrows suggest potential effects in the decoding of the NFs signal.

Recently, Wang *et al*. (2022) have brought a new piece into the puzzle that can explain how the activity of NIN is modulated during root nodule symbiosis. To understand the transcriptional regulatory mechanism of *GmNINa* in soybean, Wang *et al*. (2022) performed a yeast two‐hybrid screening to identify the interacting partner of GmNSP1a, one of the TFs required for the expression of *GmNINa* (the second ortholog of *MtNIN* in soybean). This analysis led to the identification of GmNAC181 as an interacting partner of GmNSP1a (Wang *et al*., 2022). GmNAC181 is a NAC TF whose expression significantly increases during nodule development and reaches its highest level of expression at 28 d postinoculation. To confirm its potential role in root nodule symbiosis, the authors overexpressed and knocked down *GmNAC181* in transgenic roots of soybean composite plants. Wang *et al*. (2022) observed that the overexpression of *GmNAC181* resulted in a significant increase in the number of nodules, whereas gene silencing showed the opposite phenotype. These data support the role of the TF GmNAC181 in nodule formation in soybean under normal conditions (Fig. 1a).

The fact that the expression pattern of *GmNINa* is similar to that of *GmNAC181* led the authors to hypothesize that GmNAC181 might regulate *GmNINa* expression through a transcriptional mechanism. To verify this hypothesis, first, Wang *et al*. (2022) confirmed that GmNAC181 is a nuclear‐localized protein and is a transcriptional activator in the yeast system; furthermore, it was dissected that the C‐terminal of GmNAC181 is required for its transcriptional activity. An in‐depth localization study revealed that GmNAC181 was localized in the cytoplasm, nucleus, and plasma membrane, suggesting that GmNAC181 may simultaneously exist at more than one subcellular localization. Analysis of the promoter sequence of *GmNINa* indicates that it contains 19 NAC binding sites, which strongly indicates that GmNAC181 may bind over the *GmNINa* promoter. Indeed, chromatin immunoprecipitation, electrophoretic mobility shift assay, and transactivation assays confirmed that GmNAC181 binds to the promoter of *GmNINa* and activates its expression. Altogether, these data confirm that GmNAC181 is a TF required for the transcriptional activation of *GmNINa* (Fig. 1a). Furthermore, these data raise the question of whether NAC181 is part of the large TF complex (CYCLOPS‐DELLA‐NSP2/1) required to activate the expression of NIN in other legumes or is an exclusive mechanism in soybean. If the last scenario is the case, it will be important to understand whether the four copies of *NIN* present in the soybean genome are under the same transcriptional regulation (Fu *et al*., 2021).

## Involvement of NAC181 in the regulation of salt tolerance in soybean nodulation

It is worth noting that the *GmNAC181* gene is highly induced by salt stress along with other abiotic stresses such as drought stress. The overexpression of *GmNAC181* significantly improves salt tolerance in *Arabidopsis thaliana* and soybean plants (Hao *et al*., 2011). Wang *et al*. (2022) speculated that GmNAC181 may have a more important role to play in nodulation under salt stress in soybean plants. This hypothesis was supported by the presence of both salt‐stress responsive and nodule‐specific *cis*‐regulatory elements in the promoter of *GmNAC181*. To test this hypothesis, the expression of *NAC181* was analyzed in roots inoculated with rhizobia under control and salt stress conditions. *GmNAC181* expression was reduced in the early stages of rhizobial inoculation under normal conditions, but salt stress significantly increased *GmNAC181* expression in rhizobia‐infected roots and nodules (Wang *et al*., 2022). Furthermore, *GmNAC181* knockdown dramatically decreased the number of nodules, while *GmNAC181* overexpression maintained normal root nodule numbers in salt stress. Wang *et al*. (2022) demonstrated that GmNAC181 improves root nodulation to withstand salt stress through the upregulation of *GmNINa* and other downstream nodulation genes. This implies that GmNAC181 is necessary for the development of root nodules under salt stress (Fig. 1b). The fact that GmNSP1a is phosphorylated by GmSK2‐8 under salt‐stress conditions, and that GmNAC181 is an interactor of GmNSP1a, raises the question of whether the GmNSP1a–GmNAC181 interaction is affected under salt‐stress conditions, or whether GmNAC181 acts independently to activate the symbiotic pathway and form fewer nodules under this abiotic condition.

## The potential interplay of salinity and the autoregulation of nodulation pathway

Legumes control the number of nodules through the activation of the autoregulation of nodulation (AON) pathway (Ferguson *et al*., 2019). The rhizobia‐induced CLE (RIC) peptides RIC1 and RIC2 and the Kelch repeat‐containing F‐box protein Too Much Love (TML) are key genetic components of this pathway required to restrict nodule formation (Suzuki *et al*., 2008; Takahara *et al*., 2013; Ferguson *et al*., 2014).

Mounting evidence indicates that nitrate inhibits root nodule symbiosis through activation of the AON pathway (Ferguson *et al*., 2019). Recent evidence indicates that phosphate deficiency reduces the number of nodules by activating the AON pathway (Isidra‐Arellano *et al*., 2020). The fact that salinity reduces the number of nodules in diverse legumes, it is tempting to speculate that salinity might activate the AON pathway to restrict nodule formation under this abiotic condition. To prove this, it will be necessary to evaluate whether salt stress activates the main AON genetic components (i.e. *RIC1/2* and *TML*) as observed in phosphate deficiency (Isidra‐Arellano *et al*., 2020). Additionally, it will be also interesting to test whether the reduction in the number of nodules is not observed in *ric1*, *ric2*, and *tml* mutant plants. With all this evidence, we will be better positioned to understand how salinity reduces the number of nodules and to demonstrate whether the AON pathway is required or not.

## Concluding remarks and future perspectives

Overall, our knowledge about the genetic mechanisms underlying the establishment of root nodule symbiosis under salt‐stress conditions has improved in the past 2 yr. We have learned that the modulation of the temporal *NIN* expression and NIN activity is crucial in shaping root nodule symbiosis according to salt levels in the soil. The phosphorylation of GmNSP1 by the kinase GmSK2‐8 is determinant in regulating the transcriptional activity of *NIN* in this abiotic condition in soybean. Current knowledge indicates that GmNAC181 is a crucial regulator of the expression of *GmNINa* under normal and salt‐stress conditions. These recent data also raise new questions that must be experimentally addressed to gain a deeper understanding of the molecular mechanisms underlying the adaptation of root nodule symbiosis to salt‐stress conditions. For example, it is important to explore whether the subcellular localization of GmNAC181 is subject to salt stress, and how the change in subcellular localization of GmNAC181 influences its transcriptional activity in the root and root nodules under salt stress. Furthermore, the involvement of GmNAC181 in the regulation of nodulation in other abiotic stresses remains to be explored. Along with this, it is of interest to determine whether the interactions between GmNAC181 and GmNSP1 could have an impact on the formation of the TF complex CYCLOPS‐DELLA‐NSP2, the major transcriptional machinery required for the activation of *NIN*. Future research should focus on the extent to which these pathways are conserved in other crop legumes like chickpea and *Phaseolus vulgaris*. Finally, because *Bradyrhizobium diazoeficiens* USDA 110, the strain used in the two main manuscripts discussed in this viewpoint, is hypersensitive to salt stress, it is imperative to use rhizobia strains isolated from saline soil to perform the same experiments in soybean and other legumes to confirm the relevance of NSP1, NAC18, and SK2‐8 in nodulation under salt stress. Translation of this knowledge in the creation of salt and other abiotic stresses such as drought tolerant legumes by genetic engineering any of these TFs can be breakthrough tools for agriculture in the field.

## Competing interests

None declared.

## Author contributions

OV‐L and JS proposed the conceptual idea of this viewpoint paper, wrote the manuscript, and generated the figure.

## Data Availability

All the information mentioned in this manuscript is available in the main text.
